# Comparison of Tacrolimus and Infliximab for Acute Severe Ulcerative Colitis in Hospitalized Adults: A Retrospective Study

**DOI:** 10.1111/1751-2980.70055

**Published:** 2026-06-15

**Authors:** Neta Sror, Natalie Tamir‐Degabli, Daniel Shaham, Anna Rozenfeld, Haim Leibovitzh, Ayal Hirsch, Tamar Thurm, Yulia Ron, Sigal Fishman, Nitsan Maharshak, Nathaniel A. Cohen

**Affiliations:** ^1^ Department of Internal Medicine B Tel Aviv Sourasky Medical Center Tel Aviv Israel; ^2^ Faculty of Medical and Health Sciences Tel Aviv University Tel Aviv Israel; ^3^ IBD Center, Department of Gastroenterology and Liver Diseases Tel Aviv Sourasky Medical Center Tel Aviv Israel

**Keywords:** acute severe ulcerative colitis, infliximab, salvage therapy, steroid‐refractory, tacrolimus

## Abstract

**Objectives:**

Comparative data on infliximab (IFX) and tacrolimus as salvage therapies for intravenous glucocorticoid (ivGC)‐refractory acute severe ulcerative colitis (ASUC) are limited. We aimed to evaluate the effectiveness and safety of IFX versus tacrolimus in hospitalized patients with ivGC‐refractory ASUC.

**Methods:**

This retrospective study was conducted between 2017 and 2024 at a tertiary medical center, including inpatients with ASUC who failed ivGC and received IFX or tacrolimus. The primary outcome was colectomy‐free clinical response at discharge.

**Results:**

Thirty‐seven patients received IFX (median age 27 years; 32.4% males), and 24 received tacrolimus (median age 32 years; 41.7% males). Compared with the patients who received IFX, those receiving tacrolimus had a longer disease duration (4 years vs. 1 year, *p* = 0.031) and a higher rate of prior exposure to advanced therapy (anti‐tumor necrosis factor agent: 58.3% vs. 2.7%, *p* = 1.05 × 10^−6^; Janus kinase inhibitors: 20.8% vs. 0%, *p* = 0.007). The length of hospitalization was significantly longer among those receiving tacrolimus (median 19.5 days vs. 11.0 days, *p* = 0.019). Colectomy‐free clinical response rate at discharge (81.1% vs. 83.3%, multivariate analysis *p* = 0.956) and total colectomy rate in the following year (12.5% vs. 14.3%, *p* = 0.851) did not significantly differ between the IFX and tacrolimus groups. Safety profile during hospitalization was comparable between groups.

**Conclusions:**

In ivGC‐refractory ASUC cases, IFX and tacrolimus showed comparable effectiveness and safety. Similar outcomes were observed despite a more treatment‐refractory profile in the tacrolimus group. These findings support the use of either agent as a feasible salvage option in this clinical setting.

## Graphical abstract

Colectomy‐free clinical response rates at discharge and at 1‐year follow‐up were comparable in patients with acute severe ulcerative colitis treated with infliximab or tacrolimus as salvage therapies, as shown in this retrospective study. The safety profile was comparable between groups, although the length of hospitalization was longer among tacrolimus‐treated patients, supporting both as viable options.

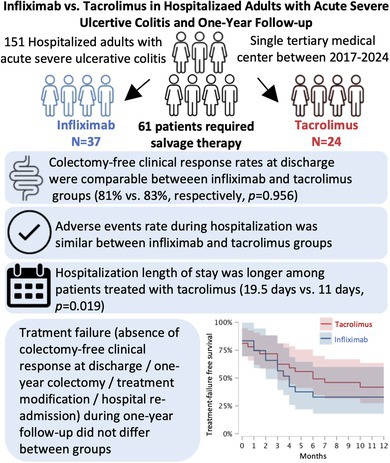

## 1 Introduction

Ulcerative colitis (UC) is a chronic immune‐mediated inflammatory disease that primarily affects the colon. Up to half the patients with UC require at least one hospitalization due to disease‐related complications, which are closely associated with disease duration and extent [1]. Approximately 25% of patients with UC may develop an episode of acute severe UC (ASUC); among them, 30% are refractory to intravenous glucocorticoids (ivGC) and require salvage therapy, while 15%–20% of them receive colectomy on their first admission [2, 3].

Salvage therapy with intravenous (IV) administration of calcineurin inhibitor (CNI), such as cyclosporine, and the addition of anti‐tumor necrosis factor (TNF)‐α agent (e.g., infliximab [IFX]) during the past two decades have significantly changed the therapeutic paradigm in ASUC [4]. Multiple studies have demonstrated the effectiveness and relative safety of these salvage therapies for ASUC, reserving colectomy mainly to nonresponsive patients [5, 6, 7, 8]. Despite their efficacy, salvage therapies present notable challenges and a considerable failure rate of 20%–30% [5]. This has provided the impetus to find other therapeutic solutions for ASUC, including oral CNI—tacrolimus—which may overcome some of the limitations of cyclosporine, including inconvenient, continuous IV administration, difficulty in converting IV to oral dosing and maintaining appropriate serum levels, and potential toxicity. Growing evidence supports the potential effectiveness of tacrolimus in patients with ASUC who are refractory to steroids [9, 10, 11]. More recently, the utility of Janus kinase (JAK) inhibitors for ASUC has also been explored [5, 12].

To our knowledge, only a few real‐world studies have compared the therapeutic outcomes of IFX and tacrolimus among patients with ASUC, showing comparable short‐ and long‐term colectomy rates and safety profiles between IFX and tacrolimus [13, 14, 15]. However, these studies included both hospitalized patients and outpatients, some of whom were defined as “steroid‐dependent” rather than strictly “steroid‐refractory.” In addition, key in‐hospital outcomes, including colectomy rates during hospitalization, have not been reported. Given that these studies were published during 2014 and 2017, which was prior to the current era of advanced treatment options, there is an urgent, renewed need to reassess post‐ASUC hospitalization outcomes, such as treatment persistence and treatment failure‐free survival. More recent comparative studies remain limited and do not focus exclusively on hospitalized patients with ivGC‐refractory ASUC. Moreover, some studies excluded those with prior exposure to advanced therapies or included only a small proportion of patients who received IFX or tacrolimus (23%) [16, 17, 18]. Selection of appropriate salvage therapy for individual patients with ivGC‐refractory ASUC remains challenging in clinical practice, as current guidelines are established based on low‐ to moderate‐quality evidence [4]. Certain factors may guide therapeutic choice. For example, though IFX is preferred because of its better clinical response and ease of administration, together with lower monitoring requirements and favorable safety profile, CNIs may be more appropriate for cases who have failed prior anti‐TNF therapy [5]. Furthermore, small molecules such as JAK inhibitors—tofacitinib and upadacitinib—are preferred in the presence of hypoalbuminemia, the need for rapid therapeutic response, and the desire to avoid immunogenicity [19]. However, there is little data to support this clinical decision‐making approach, and insights into therapeutic selection in the setting of ASUC are required.

In this study, we aimed to compare the effectiveness of IFX and tacrolimus for the treatment of hospitalized adult patients with ivGC‐refractory ASUC. The primary outcome was colectomy‐free clinical response at discharge. Additional objectives included a safety comparison during hospitalization and evaluation of their long‐term effectiveness over 1 year after discharge.

## 2 Patients and Methods

### 2.1 Study Design and Patient Enrollment

This was a retrospective, single‐center study conducted at a large tertiary care hospital (Tel Aviv, Israel) that included consecutive hospitalized patients with ASUC who were unresponsive to ivGC and required salvage therapy with IFX or tacrolimus between February 2017 and Decemberr 2024. Only those with ASUC presenting either a flare of their established UC or initial UC presentation were eligible for inclusion. ASUC was diagnosed according to the Truelove and Witts criteria, and disease extent was classified based on the Montreal classification [16, 20]. Nonresponsiveness to ivGC was determined between the third and fifth day of hospitalization based on the Oxford criteria [21]. Salvage therapy was chosen at the discretion of the treating physician, considering the clinical characteristics of the patients including comorbidities, body mass index (BMI), prior anti‐TNF exposure, and patient preference. Patients who received salvage therapy with cyclosporine or JAK inhibitors were excluded from the analysis.

The patients were further categorized into different treatment groups based on the salvage therapy they received (IFX or tacrolimus). Each patient was included in the group corresponding to the treatment they were discharged with or following colectomy after salvage treatment failure. The safety analysis included all patients who received at least one dose of either therapy during hospitalization. If a patient was treated with both therapies, both treatments were included in the safety analysis. For example, two of our patients experienced allergic reactions to IFX, resulting in treatment discontinuation and a switch to tacrolimus. Consequently, they were included in the tacrolimus group for effectiveness analysis and in both groups for safety analysis.

### 2.2 Treatment Regimens

The ivGC therapy, as indicated for ASUC, was administered IV with either hydrocortisone (Pfizer Inc., Lake Forest, IL, USA) 100 mg three times daily or methylprednisolone (Pfizer Inc., New York, NY, USA) 62.5 mg once daily. Standard induction protocols for IFX and tacrolimus were then followed [13, 14]. In brief, IFX infusion (Janssen Biotech Inc., Pennsylvania, USA) was administered at a dosage of 5 mg/kg during the hospitalization (Week 0), with the following dose of 5 mg/kg at Week 2 as part of the standard induction regimen. In select cases, based on clinical judgment, dosage escalation to 10 mg/kg or a shortened interval (administering the second dosage 1 week later) during the hospitalization was permitted.

The oral dosage of tacrolimus (Astellas Pharma US Inc., Northbrook, IL, USA) was initiated at 0.05–0.1 mg/kg twice daily. Serum trough levels of tacrolimus were measured 48 h after the first dose. Its dosage was adjusted to maintain a whole‐blood concentration within the target range of 10–15 ng/mL. A lipid profile was obtained before initiating tacrolimus administration, and serum electrolytes, particularly potassium and magnesium, were monitored daily.

### 2.3 Outcomes and Definitions

The primary outcome was the comparison of colectomy‐free clinical response rates at discharge among patients with ivGC‐refractory ASUC who received salvage therapy with IFX or tacrolimus. Clinical response was defined as a reduction of at least 50% in the patient‐reported outcome 2 (PRO2) score, which is the sum of the weighted daily stool frequency and rectal bleeding of the Mayo score at discharge [12, 22].

Secondary outcomes included post‐hospitalization colectomy rate, length of hospitalization, and hospital re‐admissions due to UC. Time to clinical response for each salvage therapy during the index hospitalization was defined as the time interval from the first day following the initiation of salvage therapy on which PRO2 score and both stool frequency and C‐reactive protein (CRP) levels decreased by at least 50%. Patients who did not achieve clinical response during hospitalization or underwent colectomy were then excluded from the analysis. A safety analysis was conducted to evaluate the rate of adverse events (AEs) during the hospitalization for ASUC.

The 1‐year treatment persistence of advanced therapy was defined as maintaining clinical response with the same agent initiated at the index hospitalization for IFX, or at the first post‐discharge physician visit for tacrolimus. These long‐term outcomes were assessed during a 1‐year follow‐up post‐discharge. Treatment failure during the 52‐week follow‐up was defined as any of the following: (i) absence of colectomy‐free clinical response at discharge; (ii) need for post‐hospitalization colectomy; (iii) UC‐related readmission; or (iv) therapeutic modification. Steroid‐free clinical remission was assessed by the treating physician at the last follow‐up visit.

### 2.4 Statistical Analysis

Continuous variables were presented as mean ± standard deviation for parametric data and median and interquartile range (IQR) for non‐parametric data. Normality of continuous variables was assessed using the Shapiro–Wilk test and visual inspection of histograms. The differences in continuous variables between groups were compared with an independent samples *t*‐test or Mann–Whitney *U*‐test, when appropriate. Categorical variables were presented as numbers and proportions or frequencies, and were compared using the Chi‐square test. Univariate logistic regression analysis was performed to compare between groups with respect to binary outcomes, such as colectomy rates and steroid‐free clinical remission at the last follow‐up visit. Multivariate analysis was performed for the major outcomes, including colectomy‐free clinical response at discharge, treatment failure at 1‐year follow‐up, and AE rates, as well as for secondary outcomes that were statistically significant (*p* < 0.05) in univariate analysis. Multivariate analysis was adjusted for age at admission, prior advanced therapy, smoking status, and ulcerative colitis endoscopic index of severity (UCEIS) [23] at admission. As patients treated with tacrolimus showed a more severe disease profile than those treated with IFX, a propensity score adjustment for treatment selection was also performed for the analysis of primary outcome to address potential confounding by indication. Given the relatively small sample size and prior literature [18], four covariates were chosen regarding treatment preference, namely age at admission, prior exposure to advanced therapeutic agents, albumin levels upon admission, and UCEIS score. A propensity score for receiving tacrolimus compared with IFX was estimated using logistic regression and was subsequently included as an adjustment variable in the multivariable model for the primary outcome. Post‐discharge treatment failure‐free survival and time to clinical response were analyzed using the Kaplan–Meier method and compared between groups using the log‐rank test. Patients who did not experience treatment failure during the study period or were lost to follow‐up were censored at their last known follow‐up date. A *p* < 0.05 was considered statistically significant. All the statistical analyses were performed using SPSS Statistics version 31.0 (IBM, Armonk, NY, USA).

## 3 Results

### 3.1 Study Population

**FIGURE 1:**
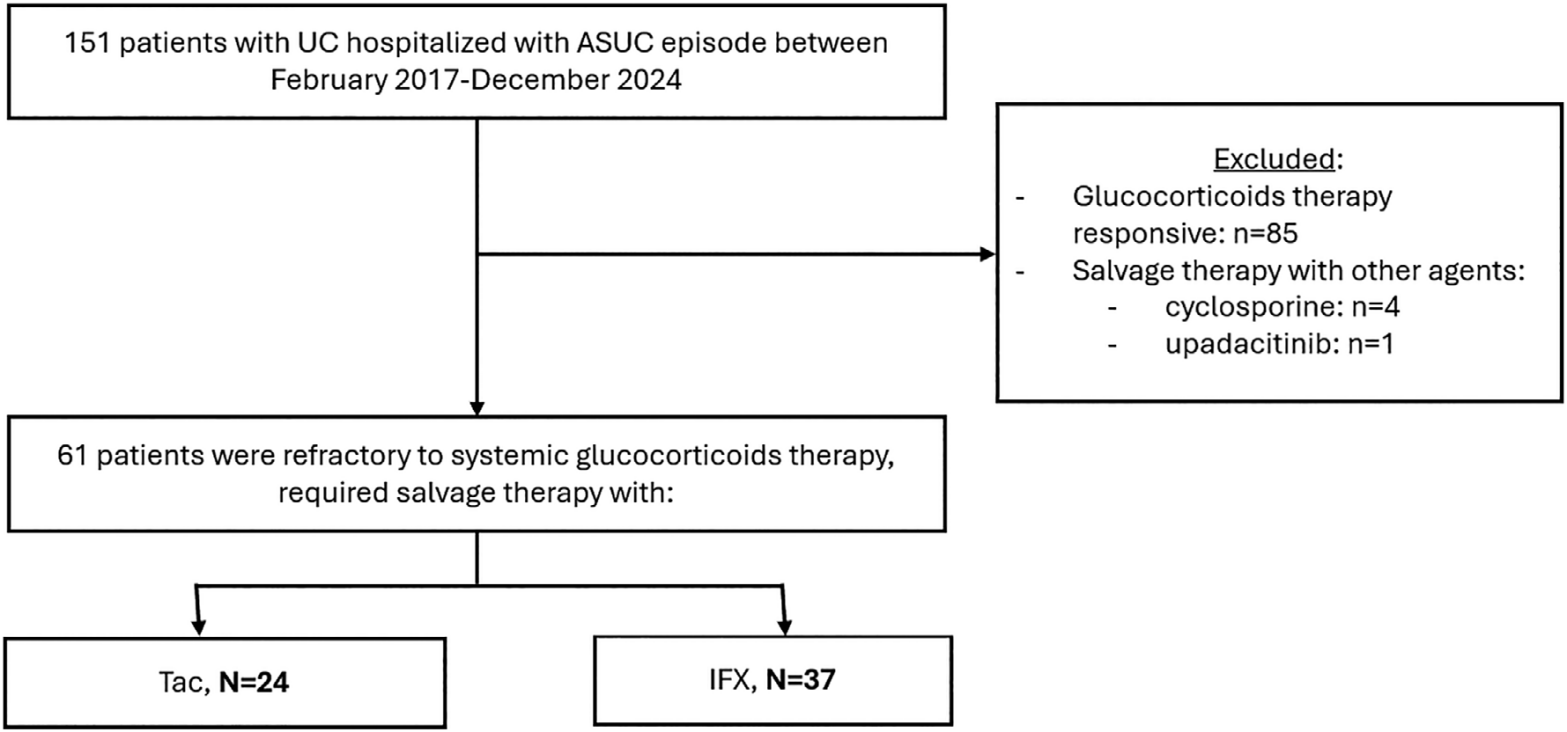
Study flowchart of patient enrollment. Patients hospitalized for acute severe ulcerative colitis (ASUC) between February 2017 and December 2024 receiving salvage therapy with infliximab (IFX) or tacrolimus (Tac) due to refractoriness to systemic glucocorticoids. UC, ulcerative colitis.

Between February 2017 and December 2024, 151 patients with ASUC were hospitalized at our tertiary medical center. Among them, 66 (43.7%) patients were refractory to ivGC and required salvage therapy, including 37 (56.1%) patients who received IFX and 24 (36.4%) who received tacrolimus and were included in the comparative effectiveness and safety analyses. The other five patients were excluded from analysis because they were treated with other salvage therapies (cyclosporine in 4 patients and upadacitinib in one). Flowchart of patient inclusion is shown in Figure 1.

### 3.2 Baseline Characteristics of Patients

Baseline characteristics of patients receiving IFX or tacrolimus as salvage therapy are summarized in Table 1. The median age at admission of the IFX and tacrolimus groups was 27 years (IQR 22–38 years) and 32 years (IQR 25.75–49.25 years), respectively (*p* = 0.326). Both groups had a female predominance (IFX vs. tacrolimus: 67.6% vs. 58.3%, *p* = 0.463). Those treated with IFX had a shorter median disease duration (1 year [IQR 0–4 years] vs. 4 years [IQR 1.75–10 years], *p* = 0.031). In addition, a higher proportion of IFX‐treated patients were never smokers (88.9% vs. 62.5%, *p* = 0.015). Disease extent was comparable between the two groups, with most patients having extensive colitis (IFX vs. tacrolimus: 54.1% vs. 70.8%, *p* = 0.19). Over half the patients treated with tacrolimus had prior advanced therapy with anti‐TNF agents (58.3% vs. 2.7%, *p* = 1.05 × 10^−6^) or JAK inhibitors (20.8% vs. 0%, *p* = 0.007). Among the 12 patients who had ASUC as the initial presentation of their UC, 10 (83.3%) were treated with IFX, while the other 2 (16.7%) received tacrolimus (*p* = 0.073).

**TABLE 1:** Baseline characteristics of patients treated with infliximab (IFX) or tacrolimus as salvage therapy for acute severe ulcerative colitis (ASUC).

Variables	IFX (*N* = 37)	Tacrolimus (*N* = 24)	*p* value
Male sex (*n*, %)	12 (32.4)	10 (41.7)	0.463
Age at admission, years (median [IQR])	27 (22–38)	32 (25.75–49.25)	0.326
Disease duration, years (median [IQR])	1 (0–4)	4 (1.75–10)	**0.031**
BMI, kg/m^2^ (mean ± SD)	22.0 ± 3.6	20.8 ± 3.8	0.258
Smoking status (*n*, %)	***N* = 36**	***N* = 24**	
Never	32 (88.9)	15 (62.5)	**0.015**
Past	2 (5.6)	6 (25.0)	0.05
Current	2 (5.6)	3 (12.5)	0.38
Comorbidities (*n*, %)			
Cardiovascular disorder	2 (5.4)	1 (4.2)	0.66
Metabolic disorder	5 (13.5)	3 (12.5)	0.958
Solid tumor	0 (0)	1 (4.2)	0.38
Hematologic disorder	4 (10.8)	1 (4.2)	0.64
Inflammation	2 (5.4)	0 (0)	0.52
Dermatologic disorder	5 (13.5)	3 (12.5)	1.00
Respiratory disorder	3 (8.1)	3 (12.5)	0.66
Others^a^	1 (2.7)	3 (12.5)	0.153
Pregnancy (*n*, %)	4 (10.8)	1 (4.2)	0.64
Disease extent (*n*, %)			
E1 (proctitis)	3 (8.1)	0 (0)	0.272
E2 (left‐sided colitis)	14 (37.8)	7 (29.2)	0.48
E3 (extensive colitis)	20 (54.1)	17 (70.8)	0.19
EIM (*n*, %)	10 (27.0)	3 (12.5)	0.176
Dermatology	5 (13.5)	1 (4.2)	0.388
Articular	3 (8.1)	2 (8.3)	1.00
Ocular	2 (5.4)	0 (0)	0.51
Past GI surgery (*n*, %)	1 (2.7)	1 (4.2)	0.754
Previous treatment (*n*, %)			
Steroids	17 (45.9)	19 (79.2)	0.01
PO	12 (32.4)	14 (58.3)	
Topical	4 (10.8)	1 (4.2)	
Both	1 (2.7)	4 (16.7)	
Antibiotics	7 (18.9)	12 (50.0)	**0.01**
5‐ASA (*n*/*N*, %)	24/36 (66.7)	18/22 (81.8)	**0.21**
Purine analogue	5 (13.5)	8 (33.3)	0.065
Advanced treatment^b^	5 (13.5)	15 (62.5)	**6.85 × 10** ^**−5**^
Anti‐TNF agents	1 (2.7)	14 (58.3)	**1.05 × 10** ^**−6**^
IFX	1 (2.7)	9 (37.5)	
Over one anti‐TNF agent	0 (0)	5 (20.8)	
Vedolizumab	4 (10.8)	7 (29.2)	0.093
Janus kinase inhibitors	0 (0)	5 (20.8)	**0.007**
Tacrolimus	0 (0)	1 (4.2)	0.39
No previous treatment	7 (18.9)	3 (12.5)	0.72

*Note*: Bold characters indicate statistical significance.

Abbreviations: 5‐ASA, 5‐aminosalicylic acid; BMI, body mass index; EIM, extraintestinal manifestations; GI, gastrointestinal; IQR, interquartile range; PO, per os; SD, standard deviation.

^a^ Other comorbidities indicate cognitive decline in the IFX group, as well as fibromyalgia, autism, and anxiety in the tacrolimus group.

^b^ Advanced treatment includes biologic treatments (anti‐tumor necrosis factor [TNF] antibodies, anti‐integrins), small molecules (Janus kinase inhibitors), etc.

Clinical parameters and details of salvage therapy are summarized in Table 2. Consistent with prior treatments, a greater proportion of patients treated with tacrolimus received advanced therapies upon admission when compared with those receiving IFX (45.8% vs. 21.6%, *p* = 0.046). Clinical presentations on admission were similar between the two groups. The median daily stool frequency was 10 times in both groups. The CRP (IFX vs. tacrolimus: 70.0 mg/L [IQR 27.5–132.7 mg/L] vs. 53.7 mg/L [IQR 22.3–167.0 mg/L]) and albumin levels (IFX vs. tacrolimus: ([32.78 ± 5.30] g/L vs. [31.80 ± 6.30] g/L) were comparable between the two groups (all *p* > 0.05). However, UCEIS was significantly higher among those treated with tacrolimus (median score: 6 [IQR 5–7] vs. 5 [IQR 4–6], *p* = 0.014). Salvage therapy with tacrolimus was initiated earlier than IFX (day of initiation: 3.5 [IQR 2–6] vs. 6 [IQR 3.5–8.5], *p* = 0.01).

**TABLE 2:** Characteristics of patients at admission, including current treatment, clinical parameters, and salvage therapeutic properties.

Variables	IFX (*N* = 37)	Tacrolimus (*N* = 24)	*p* value
Current treatment (*n*, %)			
Steroids	23 (62.2)	13 (54.2)	0.535
IV	1 (2.7)	1 (4.2)	
PO	21 (56.8)	11 (45.8)	
Topical	1 (2.7)	1 (4.2)	
Antibiotics	9 (24.3)	3 (12.5)	0.334
5‐ASA	19 (51.4)	8 (33.3)	0.166
Purine analogues	1 (2.7)	1 (4.2)	1.00
Advanced therapy^a^	8 (21.6)	11 (45.8)	**0.046**
Anti‐TNF agents	2 (5.4)	4 (16.7)	0.15
IFX	2 (5.4)	2 (8.3)	
ADA	0 (0)	2 (8.3)	
Vedolizumab	5 (13.5)	6 (25.0)	0.254
Janus kinase inhibitors	1 (2.7)	1 (4.2)	1.00
Clinical variables			
Frequency of daily defecation (median [IQR])	10 (8–20)	10 (10–15)	0.96
Body temperature, °C (median [IQR])	36.8 (36.5–37.1)	37 (36.72–37.1)	0.153
Heart rate, bpm (mean ± SD)	97.70 ± 17.80	92.95 ± 19.00	0.324
Hemoglobin, g/L (mean ± SD)	116.0 ± 13.7	110.0 ± 20.0	0.215
WBC, ×10^9^/L (mean ± SD)	12.3 ± 11.8	12.2 ± 11.95	0.953
PLT, ×10^9^/L (median [IQR])	385.00 (318.50–500.50)	455.00 (365.25–552.00)	0.126
CRP, mg/L (median [IQR])	70.0 (27.5–132.7)	53.7 (22.3–167.0)	0.80
Albumin, g/L (mean ± SD)	32.78 ± 5.30	31.80 ± 6.30	0.547
Potassium, mmol/L (mean ± SD)	3.73 ± 0.48	3.65 ± 0.59	0.549
Magnesium, mg/dL (mean ± SD)	1.98 ± 0.29	1.94 ± 0.35	0.65
UCEIS (median [IQR])	5 (4–6)	6 (5–7)	**0.014**
Recent fecal calprotectin, μg/g (mean ± SD)	2745 ± 2409	2390 ± 1986	0.80
PRO2 (median [IQR])	5 (5–5)	5 (4.75–5)	0.973
Salvage treatment properties (*n*, %)			
Day of initiation (median [IQR])	6 (3.5–8.5)	3.5 (2–6)	**0.01**
Day 1–Day 3	9 (24.3)	12 (50)	**0.039**
Day 3–Day 7	12 (32.4)	7 (29.2)	0.788
After Day 7	16 (43.2)	5 (20.8)	0.072
Serum tacrolimus (ng/mL) (median [IQR])			
During hospitalization		9.6 (7.05–14.85)	
At discharge		13 (11.2–14.9)	
Salvage treatment duration and dosing			
Duration, days (median [IQR])		14 (8–19)	
Two doses or more (*n*, %)	10 (27.0)		
Dosing at 10 mg/kg (*n*, %)	7 (18.9)		

*Note*: Bold characters indicate statistical significance.

Abbreviations: 5‐ASA, 5‐aminosalicylic acid; ADA, adalimumab; bpm, beats per minute; CRP, C‐reactive protein; IFX, infliximab; IQR, interquartile range; IV, intravenous; PLT, platelet; PRO2, patient‐reported outcome 2; PO, per os; SD, standard deviation; UCEIS, ulcerative colitis endoscopic index of severity; WBC, white blood cell.

^a^ Advanced treatment includes biologic treatments (anti‐tumor necrosis factor [TNF] agents, anti‐integrins), small molecules (Janus kinase inhibitors), etc.

### 3.3 Colectomy‐Free Clinical Response at Discharge and 1‐Year Colectomy Rate

Colectomy‐free clinical response rates at discharge were comparable between patients treated with IFX and tacrolimus (IFX vs. tacrolimus: 81.1% vs. 83.3%, *p* = 0.823) (Table 3). This was further confirmed by multivariate analysis, with an adjusted odds ratio (aOR) of 0.951 (95% confidence interval [CI] 0.156–5.800; *p* = 0.956). After the propensity score adjustment was performed, there were no significant differences between groups for colectomy‐free clinical response rates at discharge as well (95% CI 0.300–7.040; *p* = 0.645). In‐hospital colectomy rate was comparable between the IFX and tacrolimus groups (IFX vs. tacrolimus: 8.1% [3 of 37] vs. 4.2% [1 of 24], *p* = 0.55). The total colectomy rate within the following year remained comparable between groups (IFX vs. tacrolimus: 12.5% [4 of 32] vs. 14.3% [3 of 21], *p* = 0.85). A subgroup analysis of biologically naive patients in both groups showed similar colectomy‐free clinical response rates (26 [81.3%] vs. 9 [100%], *p* = 0.160).

**TABLE 3:** Comparison of clinical outcomes between infliximab (IFX) and tacrolimus‐treated patients.

Univariate analysis			
Variables	IFX (*N* = 37)	Tacrolimus (*N* = 24)	*p* value
Colectomy‐free clinical response at discharge (*n*, %)	30 (81.1)	20 (83.3)	0.823
Total colectomy rate in‐hospitalization (*n*, %)	3 (8.1)	1 (4.2)	0.55
Total colectomy rate in the following year^a^ (*n*/*N*, %)	4/32 (12.5)	3/21 (14.3)	0.851
Hospitalization, days (median [IQR])	11.0 (8.5–17.5)	19.5 (12.25–22.0)	**0.012**
Time to clinical response, days (median [IQR])	2.5 (1.0–4.0)	3.0 (1.75–4.0)	0.246
Clinical variables at discharge			
Hemoglobin, g/L (mean ± SD)	103.5 ± 13.8	100.5 ± 16.0	0.445
CRP, mg/L (median [IQR])	16.00 (7.60–28.75)	12.70 (6.23–26.90)	0.34
Albumin, g/L (median [IQR])	32.1 (29.0–33.8)	32.0 (31.0–38.0)	0.48
PRO2 score (median [IQR])	1 (0–2.5)	1.5 (1–2)	0.973

One‐year F/U post‐discharge (*n*, %)^b^	*n* = 29	*n* = 18	
Treatment persistence	20 (69.0)	8 (44.4)	0.096
Steroid‐free clinical remission	21 (72.4)	10 (55.5)	0.236
Treatment failure^c^	19 (51.4)	15 (62.5)	0.392

Multivariate analysis^d^			
Variables	aOR	95% CI	*p* value
Colectomy‐free clinical response at discharge	0.951	0.156–5.800	0.956
Hospitalization (days)	10.50^e^	1.77–19.30	0.019
Treatment failure	2.53	0.60–10.46	0.199

*Note*: Bold characters indicate statistical significance, with the IFX group serving as the reference.

Abbreviations: aOR, adjusted odds ratio; CI, confidence interval; CRP, C‐reactive protein; F/U, follow‐up; IQR, interquartile range; PRO2, patient‐reported outcome 2; SD, standard deviation.

^a^ The analysis included 53 patients with at least 6‐month follow‐up post‐discharge (IFX: *n* = 32; tacrolimus: *n* = 21).

^b^ Compared between the advanced therapy initiated at the 30‐day follow‐up visit and at the last physician visit in a 1‐year follow‐up, after excluding patients undergoing colectomy and lost to follow‐up.

^c^ A composite outcome of any of the following: the absence of colectomy‐free clinical response at the index hospitalization, post‐discharge colectomy, re‐hospitalization, and treatment discontinuation within 1‐year follow‐up. Analysis included the entire cohort (*n* = 61).

^d^ Adjusted for age at admission, smoking status, previous advanced treatments, and ulcerative colitis endoscopic index of severity (UCEIS) score at admission.

^e^ Coefficient (additive effect).

### 3.4 Secondary Outcomes

The length of hospitalization was longer among patients treated with tacrolimus compared with those treated with IFX (19.5 days [IQR 12.25–22.0 days] vs. 11.0 days [IQR 8.5–17.5 days]; B coefficient 10.50, 95% CI 1.77–19.30, *p* = 0.019), while the rate of at least one UC‐related readmission within the following year was similar between the two groups (IFX vs. tacrolimus: 11 [29.7%] vs. 5 [20.8%], *p* = 0.44).

**FIGURE 2:**
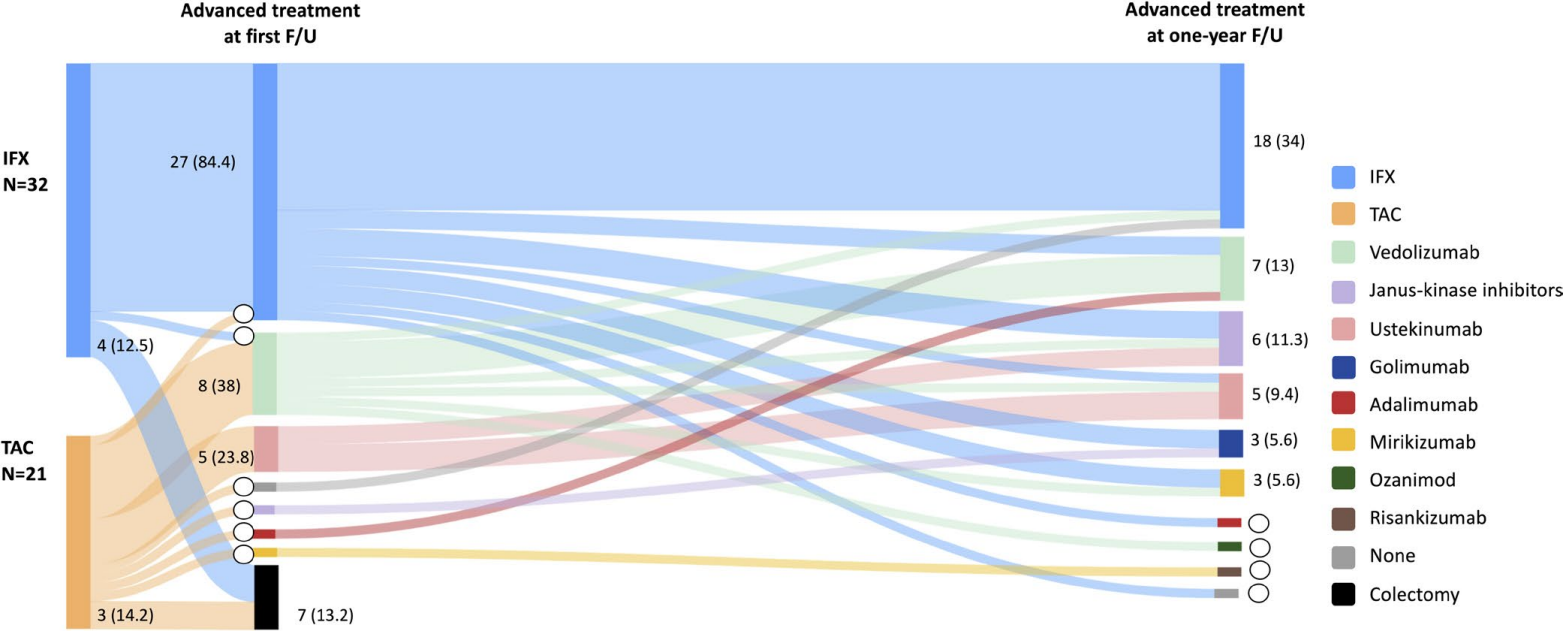
Sankey diagram illustrating flow of post‐discharge advanced treatment agents across follow‐up over a 1‐year period, as well as colectomy rates. The figure includes all patients with available follow‐up data (*n =* 53), excluding those lost to follow‐up. The diagram consists of three stages (left to right): (i) initial salvage therapy at discharge (infliximab [IFX]: *n* = 32; tacrolimus [TAC]: *n* = 21); (ii) advanced treatment initiated at the first follow‐up (F/U) visit (approximately 30 days post‐discharge); and (iii) advanced treatment at 1‐year F/U. The denominator for the second stage (advanced treatment at first F/U visit) reflects the number of patients in each salvage therapy group separately. Denominator for the third stage (advanced treatment at 1‐year F/U) and that for colectomy represent the total cohort for analysis (*n* = 53). ○ One patient was treated with this agent. Data are expressed as number and proportions.

**FIGURE 3:**
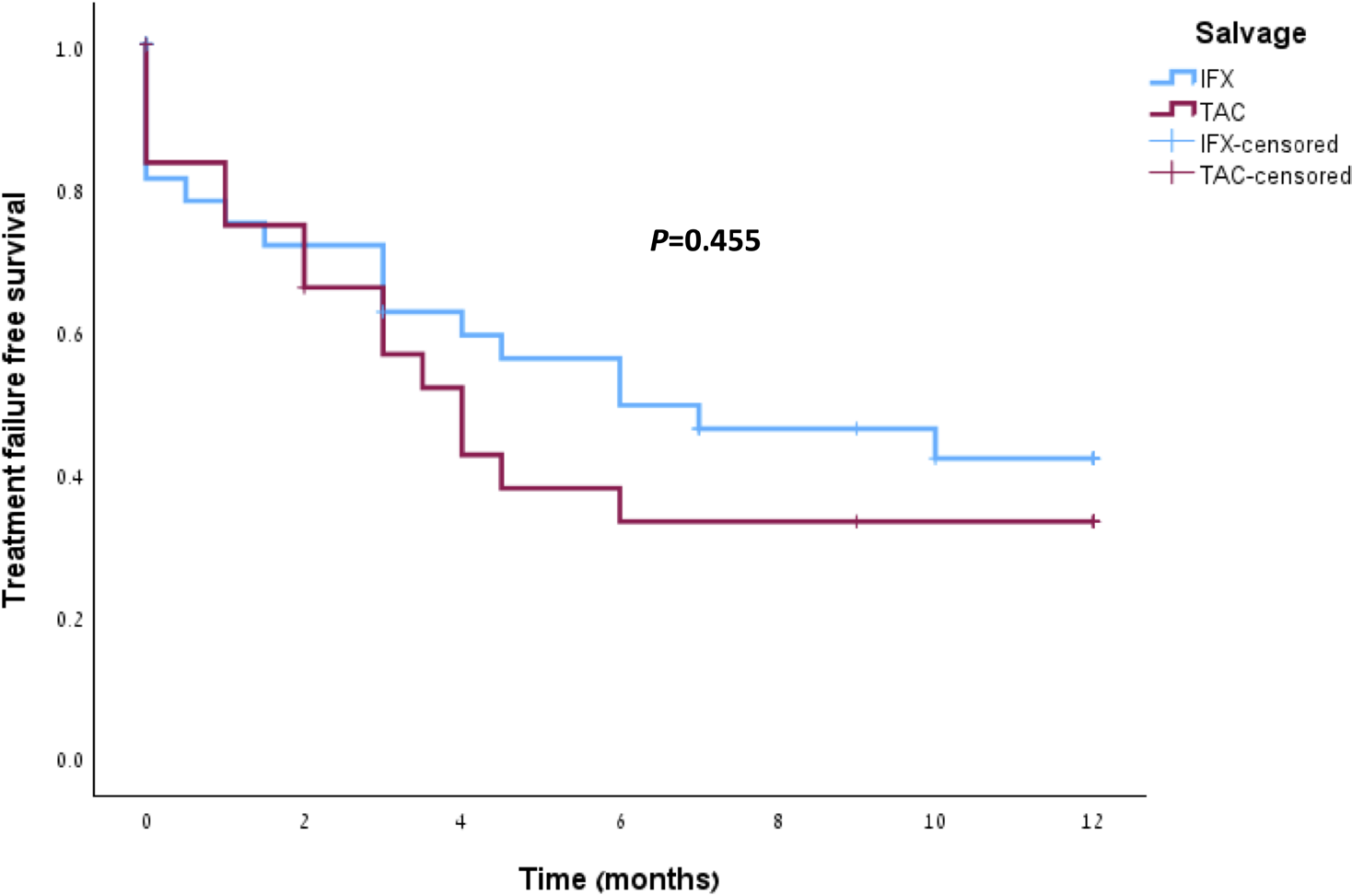
Kaplan–Meier treatment failure‐free survival curve comparing patients treated with infliximab (IFX) and tacrolimus (TAC). Treatment failure was defined as a composite outcome of any of the following: the absence of colectomy‐free clinical response at discharge, post‐discharge colectomy, re‐hospitalization, and treatment modification within 1‐year follow‐up. Time 0 represents the date of hospital discharge. *p* value was calculated using the log‐rank test.

Post‐discharge evaluation was performed in 47 patients, including 29 and 18 patients treated with IFX and tacrolimus, respectively. Fourteen patients were excluded because of the need for colectomy during hospitalization or within 30 days of discharge (*n* = 6) or lost to follow‐up post‐discharge (*n* = 8). Treatment persistence was observed in 20 (69.0%) and 8 (44.4%) patients in the IFX and tacrolimus groups, respectively (*p* = 0.096). Nine patients in the IFX group underwent advanced treatment modification, including 4 due to immunogenicity, 3 due to loss of response, one due to an AE of dyspnea, and one due to patient decision. In addition, one patient in the IFX group underwent colectomy during the following year. In the tacrolimus group, as tacrolimus was used as induction therapy, all patients were transitioned to maintenance therapy. During the follow‐up period, 10 patients required modification of their subsequent therapy. Among them, 7 patients were treated with vedolizumab (*n* = 5) or ustekinumab (*n* = 2) and later experienced loss of response. Two patients received IFX and mirikizumab, and also had loss of response. One patient received a JAK inhibitor and developed recurrent rectal bleeding after tacrolimus discontinuation. Figure 2 illustrates post‐discharge advanced treatment at the first and 1‐year follow‐up visits, as well as total colectomy rates. The figure excludes those lost to follow‐up (*n* = 8) and includes all patients with available follow‐up data (*n* = 53). Steroid‐free clinical remission, as defined by the treating physician at the last follow‐up visit, did not differ between patients treated with IFX and those receiving tacrolimus (72.4% vs. 55.5%, *p* = 0.236). The composite outcome of treatment failure did not differ between the IFX and tacrolimus groups (19 [51.4%] vs. 15 [62.5%], *p* = 0.392). No significant difference was observed in treatment failure‐free survival (*p* = 0.455), as shown in Figure 3. Further details on secondary outcomes are presented in Table 3.

### 3.5 Safety Analysis

The rate of any AEs did not differ between the two groups (IFX vs. tacrolimus: 21 [47.7%] vs. 13 [52.0%]; aOR 1.32, 95% CI 0.35–4.97, *p* = 0.675) (Table 4). Electrolyte abnormality was the most common AE, with hypomagnesemia being the most frequent AE in the tacrolimus group, which was reported by half the patients. Five (13.5%) patients treated with IFX developed infection during hospitalization, including 3 with bacterial infection and 2 with respiratory viral infection. In addition, 3 patients receiving IFX experienced allergic reactions; two of them required treatment discontinuation and switched to tacrolimus as a salvage therapy. Consequently, they were included in the tacrolimus group for the effectiveness analysis.

**TABLE 4:** Adverse events (AEs) that occurred during hospitalization in patients treated with infliximab (IFX) or tacrolimus.

AEs (*n*, %)	IFX (*N* = 37)	Tacrolimus (*N* = 24)
Any AEs (*n*/*N*, %)	21/44 (47.7)	13/25 (52.0)
Patients with at least one AE	14 (37.8)	13 (54.2)
Treatment discontinuation due to AEs	2 (5.4)	0 (0)
Allergic reactions	3 (8.1)	0 (0)
Electrolytes abnormalities	5 (13.5)	12 (50.0)
Hypokalemia	4 (10.8)	4 (16.7)
Hypomagnesemia	4 (10.8)	12 (50.0)
Elevated LFT	2 (5.4)	1 (4.2)
Infection	5 (13.5)	1 (4.2)
Bacterial^a^	3 (8.1)	0 (0)
Viral^b^	2 (5.4)	1 (4.2)
Hematologic abnormality^c^	3 (8.1)	0 (0)
Obstetrics^d^	1 (2.7)	0 (0)
Hypoalbuminemia	1 (2.7)	0 (0)
Hypertension	1 (2.7)	0 (0)

*Note*: Any AEs reflect the total number of reported AEs.

Abbreviation: LFT, liver function test.

^a^ Two patients were with upper respiratory tract infections and one patient was with enterococcus bacteremia.

^b^ One patient in the IFX group was with influenza and one patient in each treatment group had coronavirus disease 2019.

^c^ Two patients were with anemia and one patient with venous thromboembolism.

^d^ One woman was pregnant.

## 4 Discussion

In recent years, there has been a paradigm shift in the medical management of ASUC, with IFX and CNIs becoming the mainstay salvage therapies for patients admitted with ivGC‐refractory ASUC. Despite guideline recommendations detailing the approach to the treatment of ASUC, adherence to these protocols in real‐world setting remains suboptimal [2].

In the current study, we found that IFX and tacrolimus had comparable clinical effectiveness and safety in inpatients with ivGC‐refractory ASUC. Prior comparative data did not focus exclusively on this high‐risk hospitalized population [13, 14, 24], which may have limited their abilities to accurately assess effectiveness outcomes such as short‐term clinical response and colectomy prevention in cases with acute disease. In contrast, the landmark CONSTRUCT and CYSIF studies specifically compared the efficacy of IFX and cyclosporine in inpatients with ivGC‐refractory ASUC, thereby providing robust evidence for these therapies in this context [7, 8]. So far, comparable data in this high‐risk inpatient population are lacking for the comparison of IFX and tacrolimus, which is a gap our study aimed to address.

Two recent retrospective studies evaluated salvage therapies in hospitalized patients with UC but did not specifically address those with ivGC‐refractory ASUC [16, 17]. In addition, Naganuma et al. did not perform a direct comparison between IFX and tacrolimus. Therefore, to our knowledge, this is the first clinical study to directly compare IFX and tacrolimus in a well‐defined cohort of hospitalized patients with ivGC‐refractory ASUC.

In the current study, we found comparable colectomy rates between the IFX‐ and tacrolimus‐treated groups both during the index hospitalization and at 1‐year follow‐up. A subgroup analysis of biologically naive patients in both groups revealed similarly consistent outcomes. These findings align with prior studies comparing cyclosporine and IFX [8]. Takahashi et al., however, reported that tacrolimus‐treated patients with UC had a higher rate of colectomy when compared with those given IFX [17]. The reason might be that this study included overall hospitalized patients with UC, not exclusively those refractory to ivGC, and excluded individuals with prior exposure to advanced therapies. This likely reflects a less refractory population and may explain the divergent outcomes.

Compared to our patients treated with IFX, those treated with tacrolimus had a longer disease duration and were more frequently treated with advanced therapies (both prior to admission and at admission) and had a more severe disease activity as verified by the UCEIS. These differences suggest that tacrolimus may have been prescribed for patients with more severe disease or prior anti‐TNF failure. As implied, the proportion of patients who had received previous advanced therapy is the major difference between groups, and it likely influenced therapeutic decision‐making in our cohort. Furthermore, although the sample size of our study was small, tacrolimus remained effective in patients with prior JAK inhibitor treatment. These data are particularly encouraging considering the increasing use of JAK inhibitors both in outpatients and as salvage therapy for patients with ASUC [19].

Hospitalization duration was longer among patients receiving tacrolimus in the current study. Similar trends have been observed in patients with ASUC treated with cyclosporine compared to IFX, with a longer hospital stay which contributes to increased overall costs [8]. The longer hospitalization may be partially linked to a more refractory disease among the tacrolimus group, as well as the complex management required with tacrolimus.

In addition, AEs between the two groups were similar. Most AEs were mild and reversible, primarily involving electrolyte disturbances. While hypomagnesemia and hypokalemia were considered AEs only for cases that occurred following the initiation of salvage therapy, it cannot be excluded that these electrolyte abnormalities were partially attributable to ASUC‐related volume depletion. As for tacrolimus, hypomagnesemia can be explained via its renal clearance mechanism [25]. The overall safety findings are consistent with those of a previous study [14].

Strengths of this study include the evaluation of both short‐term (in‐hospital) and long‐term (1 year after hospitalization) outcomes, including colectomy rates, laboratory indicators, AEs, and treatment persistence. However, several limitations should be considered. First, the retrospective nature of our study has limited the generalizability of the results, and randomization could not be performed. Second, the small sample size might have reduced the statistical power. Third, the two groups showed differences in baseline disease severity; however, to address potential confounding factors by indication, a propensity score adjustment for treatment selection was performed. Fourth, because of the retrospective study design, important variables—potentially those affecting treatment persistence or failure, such as immunogenicity, drug level monitoring for IFX‐treated patients, and treatment adherence—could not be reliably assessed. Fifth, although the study was conducted in a tertiary care center, its single‐center setting may have limited the applicability of findings to other institutions with different patient populations and standards of care, particularly regarding colectomy rates. Sixth, safety analysis might be incomplete, as AEs were not reported in a standardized manner.

In conclusion, IFX and tacrolimus showed comparable effectiveness and safety profiles as salvage therapies for hospitalized adult patients with ivGC‐refractory ASUC. Our findings suggest that both agents remain viable treatment options for ASUC. Tacrolimus may be a valuable treatment option in patients previously exposed to advanced therapies, although it should be considered alongside its association with prolonged hospitalization. Prospective multicenter studies with large sample sizes are warranted to confirm our findings.

## Conflicts of Interest

The authors declare no conflicts of interest.

## Data Availability

The data that support the findings of this study are available from the corresponding author upon reasonable request.
